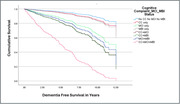# Cognitive Complaint, Mild Cognitive Impairment, and Mild Behavioral Impairment as Predictors of Dementia or Alzheimer’s Disease in a Population‐Based Sample: The Cache County Study

**DOI:** 10.1002/alz.086971

**Published:** 2025-01-03

**Authors:** Ryan C. Herd, Maddy Mills, Alexandra G. Hammond, Hector L. Gonzalez, Mikaela Drewel, Gail B. Rattinger, JoAnn T. Tschanz

**Affiliations:** ^1^ Utah State University, Logan, UT USA; ^2^ Binghamton University, Binghamton, NY USA

## Abstract

**Background:**

Mild Cognitive Impairment (MCI) and Mild Behavioral Impairment (MBI) represent transitional states between normal aging and incipient dementia. We examined whether presence of cognitive complaint (CC), MCI, and MBI differentially predicted risk for all‐cause dementia or Alzheimer’s disease (AD) in a population‐based sample.

**Method:**

Participants included 598 older adults [55% female, with mean (SD) age = 78.84 (7.51) years] without dementia at baseline of the Cache County Study on Memory in Aging. All completed a clinical assessment and were followed in subsequent waves until dementia diagnosis or non‐participation, for a maximum follow‐up time of 12 years. MCI classification was based on neuropsychological assessment with test scores falling 1.5 SD < mean on a single measure or 1.0 SD < mean on multiple measures per cognitive domain, according to aged‐based norms. CC was based on informant report of difficulties in memory, orientation, judgment, social functioning, home‐based activities, language, and recognition of others. MBI was based on the Neuropsychiatric Inventory (NPI), supplemented with informant interview. Participants were categorized as positive/negative for CC, MCI, or MBI only, or in combination; those negative on all three attributes served as the reference category. Separate Cox regression models examined CC‐MCI‐MBI status and time to all‐cause dementia/AD or right censoring, covarying age, sex, education, and APOE ε4 status.

**Result:**

One hundred fifty‐six individuals developed dementia (118 AD). Compared to the reference category, MCI only [Hazard Ratio HR (95% CI) = 3.51 (1.49 – 8.22)], CC*+*MCI [HR (95%CI) = 5.24 (2.33 – 11.79), CC+MBI [HR (95%CI) = 5.29 (1.96 – 14.32)], MCI+MBI [HR (95%CI) = 4.31 (1.24 – 14.94), and CC+MCI+MBI [HR (95%CI) = 17.11 (7.58 – 38.65)] were associated with increased risk of dementia whereas MBI only or CC only was not. For AD, results were similar, except that MCI+MBI was *not* associated with increased risk (p = .165).

**Conclusion:**

The presence of MCI with or without cognitive complaint or in combination with MBI predicted future AD or all‐cause dementia. However, MBI was predictive only in combination with cognitive complaint/MCI. These results emphasize the importance of conducting both cognitive and behavioral assessments to buttress reports of cognitive concerns.